# Transitioning diets: a mixed methods study on factors affecting inclusion of millets in the urban population

**DOI:** 10.1186/s12889-023-16872-5

**Published:** 2023-10-13

**Authors:** Suruchi Singh, Vidya Vemireddy

**Affiliations:** Centre for Management in Agriculture, Indian Institute of Management Ahmedabad, Ahmedabad, India

**Keywords:** Dietary shift, Urban affluent, Millets, Health, Social Influence, Awareness, Availability

## Abstract

**Background:**

The increasing health challenge in urban India has led to consumers to change their diet preferences by shifting away from staple cereals and making way for healthier foods such as nutri-cereals like millets and other diverse food groups. Taking the case of millets, this study seeks to uncover the exact drivers for this shift of consumers away from a traditional cereal dense diet to a nutritionally more diverse diet that includes nutri-cereal. We also look at deterrents that dissuade consumers from shifting to millets.

**Method:**

We use primary data by surveying respondents through interviews and focused group discussions and online questionnaires. A total of 20 personal consumer interviews and 4 focus group discussions having 8–12 members each were conducted to arrive at the measures for the study. We use logistic regression and Structural Equation Modeling for data analysis. Responses were obtained across major metropolitan cities and tier 2 cities of India thus ensuring representation of geographical, cultural and diet diversity. 875 participants’ responses were analysed for results.

**Results:**

Health reasons and social networks are the major drivers for shift to millets while lack of awareness, lack of easy availability, high prices, lack of branded products, family being averse to switching to millets and lack of attractive promotional cashbacks and discounts are major deterrents to trying out millets.

**Conclusions:**

Diet focussed interventions are urgently needed to curb rising diet related non communicable diseases. Government policies aimed at greater production of millets, running awareness campaigns on mass media and private sector initiatives aimed at generating better value added market offerings could lead the way.

**Supplementary Information:**

The online version contains supplementary material available at 10.1186/s12889-023-16872-5.

## Background

Globally, diet patterns are undergoing a transformation due to economic transition from subsistence to more modern systems. This transition is known as the Nutrition transition (NT) and is characterised by changes in diet patterns and physical activity [1]. It is driven by urban consumers and is accompanied by drastic changes in food production and marketing systems [2]. It is characterised by an overall decrease in cereal consumption in more urbanised and affluent regions of India [3]. Studies have shown that the prevalence of obesity among the urban population was 12.7% compared to 5.4% in rural areas of the country. A total of 42.9% of people with BMI > 25 belonged to the upper middle class in urban areas [4]. Obese and overweight women are largely concentrated in affluent Indian urban households [5]. This necessitates the need to study the dietary patterns of the affluent urban section of India.

A direct outcome of nutrition transition is a greater reliance on processed and unhealthy foods [6]. Unhealthy diets coupled with decreased physical activity have led to a spike in diet related noncommunicable diseases (DR-NCDs) [7]. They account for 71% of the total deaths worldwide [7]. Recent studies highlight the need for providing sufficient intake of healthy and environmentally sustainable diets in the wake of these developments [8, 9]. There is a call for a shift towards nutritious and sustainable foods that are part of alternative food networks [8, 10]. Consumers are becoming aware of the importance of healthy and nutritious food and are making a gradual shift towards healthy eating [11]. The affluent section of the population understands its importance and looks for high-quality food with health attributes [12].

### Role of millets

There has been a proliferation of novel food types, both indigenous and exotic, in the health food category [13]. Organic foods, foxnuts, exotic produce such as avocado, kiwi and quinoa, several novel varieties of rice such as brown rice, red rice, black rice and several others are being marketed today as superfoods [14]. Superfoods are projected to be healthier alternatives to standard food options available in the market and are usually sold unprocessed [10]. One such example of a non-mainstream but indigenous food group is millet which has the potential to enhance the health profile of consumers [15]. It is interesting to note, that India had a rich history of cultivation and consumption of millets right up to the Green Revolution. In the era preceding the Green Revolution, they were consumed widely as food and fodder. In recent years, the area under their production as well as their use as human food have drastically declined [16]. However, efforts have been made to revitalise their production and consumption. There is much effort to increase millet production and consumption at the national and international levels. This is evident from the fact that the year 2023 has been designated as the “International year of millets” [17]. Millets have piqued the interest of multiple stakeholders including consumers, farmers, food entrepreneurs, governments and policy makers because of the various benefits that they are endowed with [13, 17, 18]. Millets are rich in micronutrients, especially zinc, calcium, iron, magnesium, potassium, phosphorus, dietary fibre and critical vitamins such as thiamine, niacin, folic acid and riboflavin [19]. They are crucial to managing the risk of obesity, hypertension and micronutrient deficiency [15, 19, 20]. This speaks of the immense health potential of consuming millets. India is the world’s largest producer and consumer of millets in the world [21]^1^. Being sustainable and eco-friendly, millets are crucial to food and nutritional security [8, 22] [23, 24]. Recent trends indicate a clear shift away from staples such as rice and wheat in favour of millets, especially among the educated urban populace of the country [10]. Millets, also known as nutri-cereals, have found a shelf space in urban grocery stores today [23, 24] [17] [21] [8]. By partly replacing staple cereals with millets, consumers are increasing their dietary diversity.

### Gaps addressed by our study

Taking millets as a case, we attempt to understand this change in consumer preferences in greater detail. Through our study, we focus on understanding the factors responsible for this phenomenon. We know, from the extant literature, that at one end there are health-related concerns [25, 26] while at the other, there are marketing systems influencing these changes [2, 23]. On the one hand there are social networks, while on the other hand, there are policy frameworks driving these changes [27]. Several dimensions within these broad drivers influence the changing food habits [13]. They are complex, multifaceted constructs that need to be measured to bridge this knowledge gap. Addressing this gap in the literature is critical because knowing what drives the consumers to shift to millets can be key to increasing their uptake in our populace thereby bringing about favorable health and economic outcomes.

Despite the efforts spent on spreading awareness and increasing access to millets, there is still a section of the urban educated populace that has not yet made the shift. Knowing the reasons behind this behavior is important because of the implications it can have on human and environmental health. This study also seeks to understand the major deterrents that dissuade consumers from shifting to millets.

### Why is this study important?

Our study, with its twin objectives, has major implications for millet entrepreneurs, marketers and policymakers. It adds to the growing body of work around healthy eating and changing consumer demands and preferences in the area of health and nutrition, specifically for millets. It is a step towards better understanding the drivers and deterrents that impact consumers’ shift to millets. Another important contribution of this study is the use of mixed methods. It uses the findings obtained from the qualitative study to design and develop the survey questionnaire for the empirical study. This study also estimates latent constructs that cannot be observed directly, thereby making a process contribution to the literature. The qualitative part of this study provides a nuanced picture of the factors that influence people’s decision to switch to millets. For example, health, advertising and social media have been shown by previous studies to influence consumer choices [13, 27, 28]. However, the qualitative part of our study tells us what finer variables within the larger constructs of health, advertising and social media are responsible for these choices. This study is an amalgamation of the different variables within the ambit of health, advertising and social influence. To the best of our knowledge, this is the first study that looks at the shift to millets and not just millet consumption per se. Furthermore, it tracks actual consumption behavior and practices followed by consumers at home rather than just the choice or intention to purchase. Our study looks at the consumption of whole millets and/or their flour only as they are a natural substitute for cooked rice and flatbread. Basically, they are healthier type of grains [22] that form a major proportion of our meals. Consequently, shifting to them and hence cutting down on rice and wheat will have greater health benefits. Today, apart from regular millet grains and flour, several product variants such as crackers, cookies, pops, muffins and others are available to cater to a wider consumer base. While they do receive traction, their healthiness remains a question because of their industrial processing. Hence, this study is critical from the standpoint of the kind of food choices consumers make within the larger umbrella of healthy products. Additionally, this study strengthens the argument in favor of indigenous traditional foods at a time when the consumer mind space is being constantly bombarded with the exultation of more expensive exotic superfoods [29].

In the next section we outline the details of the data and methodology used. After a detailed presentation of the results, we discuss the implications in the final section.

## Methods

### Sample and data collection

This is a mixed methods study involving both qualitative and quantitative methods.

The first part of the study involved qualitative methods. We conducted a formative field work from December 2019 to February 2020 in Ahmedabad city of Gujarat, India to design the survey and tools. The city of Ahmedabad in Gujarat has a fairly rich dietary diversity as well as residents with regional diversity. It is the largest city in the Indian state of Gujarat and has a high degree of urbanisation [30]. People from several states of India reside here. As a result, several types of foods are easily available here. We felt that conducting the preliminary qualitative study in Ahmedabad would give us a fair picture of where our target population stands in the context of our exploration. The tools consisted of personal interviews with consumers, focus group discussions and in-store consumer surveys. A total of 20 personal consumer interviews and 4 focus group discussions with 8–12 members each were conducted. To triangulate information collected from the consumers, we also conducted interviews with 5 supermarket staff and 5 flour milling staff across 3 supermarkets and 3 milling units. Respondents were randomly selected and were asked about their availability to participate in the focus group discussions. Within each group, quotas for age, gender, income levels and education levels were set so as to achieve more or less homogenous groups of participants in all the four discussions. New topics continued to emerge during the first three rounds of discussions. During the fourth group discussion, however, no new topic emerged, thereby leading to saturation. Saturation refers to the point at which little or no new codes/categories emerge from the data [31]. Hence, we closed at four groups. Each group discussion lasted for approximately 60–75 min. A semi-structured discussion guide was used for moderating and guiding the discussion. It included questions relating to the consumption of millets as a regular part of their diet. This exercise gave us insights into the quantum, frequency and reasons for the purchase and consumption of millets. Based on these studies and the prevalence of NCDs among the urban population, a sample from this pool was chosen for the study. Great millet or sorghum (jowar), pearl millet (bajra) and finger millet (ragi) are the principal millets being consumed in India [32]. Consequently, only these three millets were included in our study although several other minor millets are produced and consumed throughout the country.

In addition to antecedents and deterrents outlined by extant literature on the topic [13], the results of this qualitative study were used to develop the survey questionnaire that formed the second part of the study involving quantitative techniques. To reach out to a diverse set of respondents, we administered the questionnaire online through a web-based platform through *Qualtrics ©* software. Prior to rolling out the survey, we conducted multiple pilots to pre-test the survey questionnaire and incorporated the changes based on the feedback received. Consumers from metropolitan and tier two cities geographically spread across India such as Delhi National Capital Region, Mumbai, Kolkata, Chennai, Bengaluru, Hyderabad, Jaipur, Patna, Ranchi, Indore, and Kochi responded to the survey. We administered the survey online because online advertising is targeted largely at educated, internet savvy, health conscious, affluent customers [33]. Furthermore, it is a time and cost effective method to reach out to a wider and geographically more dispersed audience. Our objective was to have variations across our sample on parameters such as geography, age groups, education groups, income groups, nature of job and dietary patterns. Our target sample was urban population with a minimum educational qualification of graduate and a minimum monthly household income of INR 50,000. Data were filtered accordingly. However, all the respondents fulfilled our criteria as the sampling technique used was purposive convenience sampling.

A total of 3102 responses were sought from the online survey but only 875 responses were complete and were included in this study. This yields a response rate of 28.2%. This is an acceptable response rate for online surveys as indicated by several other studies also [34, 35] that have used online surveys. Informed consent was obtained from the respondents before the start of the survey. Personal details such as name, email id and phone number of respondents were not recorded. Respondents could move to the next page only after completing all the questions on the current page. This ensured minimal data loss. The survey, on average, took 10 min to complete.

Online surveys reduce social desirability bias [36] and hypothetical bias [37].^2^ In this study we conducted the analysis on a total sample of 875 responses.

### Questionnaire and measures

The questionnaire consisted of questions to ascertain whether respondents consumed millets. Following an affirmative response, they were directed to questions to ascertain reasons for consuming millets or influencing factors. They were also asked to rate their current consumption of millets against the consumption five years ago as increased, decreased or remained constant. This allowed us to understand if there was a shift in consumption recently. We measured determinants for shift in consumption by giving them choices emanating from existing literature and discussions and personal interviews conducted during the formative fieldwork and asking them to rate them from most likely being rated 1 to least likely being rated 3. Table 1 contains a summary of these measures.

**Table 1:** Summary of measures of determinants for shift in consumption

Reasons for eating millets	Description
Been traditionally eating it	This refers to respondents who have traditionally been eating millets.
Advised by doctor due to existing health conditions (viz. hypertension, diabetes, obesity)	Patients diagnosed with lifestyle diseases are often advised by their doctors and dieticians to include more of these millets in their diet to prevent the diseases from aggravating further.
Allergic to gluten	People who suffer from gluten allergy avoid consuming wheat and wheat based products and switch to millets and rice.
Advised by doctor to pre-empt illnesses viz. hypertension, sugar etc.	People who are at the risk of contracting lifestyle diseases are often advised by doctors to suitably modify their diet and lifestyle and include millets in their diet.
Self-initiated measure to pre-empt lifestyle diseases	Health conscious people proactively follow a good diet and lifestyle to avert or delay the onset of these diseases.
In-store advertisements and inducements	Advertisements inside stores, freebies, sampling, discounts and cashbacks often serve as inducements to purchase.
Social media advertising	Social media advertising and influence is all pervasive, including in the domain of food.
Friends/relatives act as influencers	People’s social network plays a huge role in our consumption preferences.
Celebrity endorsements	Celebrities, including celebrity chefs and celebrity dieticians are also major influencers.
To cultivate healthy eating habits in family, esp. in children	Childhood dietary habits shape up health outcomes well into adulthood as well.
Environmentally sustainable	Millets are environmentally sustainable grains as they need very less water and chemicals for their growth (75) environmentally conscious consumers.

Those who answered the question on consumption in the negative were directed to measures of deterrents for not consuming millets. We measured deterrents following the same methodology as in the last paragraph and asked them to rate the deterrents from most likely being rated 1 to least likely being rated 3. Table 2 contains a summary of these deterrents.

**Table 2:** Summary of measures of deterrents for millet consumption

Reasons for not consuming millets	Description
Not much awareness about their health benefits	Not enough awareness about millets and their nutritional value
Not easily available	Not readily available in places where they are not traditionally consumed.
Existing forms are not very palatable	People look for tasty food options
Looking for organic products	Organic edible products are known to be free of chemical residues and are hence deemed healthier.
Looking for branded products	Health conscious consumers are more particular about the product attributes and brand for them is an important product attribute because it confers a feeling of trust and satisfaction.
Price point is quite high	Millets are sold at a higher price point at places where usually the urban affluent class goes to shop, namely supermarkets and online stores.
No lucrative cashbacks/discounts/free gifts	In the absence of incentives like discounts, free gifts and cashbacks, people do not want to risk trials.
I’d rather prefer quinoa and newer exotic healthy options.	Products like quinoa, teff and chia seeds are marketed as exotic products and find buyers.
Family members do not prefer consuming millets.	Since different people in the same family have different food preferences, it becomes difficult and time consuming to prepare different dishes.
Inadequate cooking skills	People who do not come from traditionally millet eating families express their inability to cook them properly.

### Data analysis

Since our study is a mixed methods study, we had conducted focus group discussions as a qualitative study technique in order to obtain deeper insights into our target group’s consumption behaviour with regard to millets. The discussions of all the sessions were transcribed using the software Google docs. Transcripts of the focus group discussions were analysed using content analysis. An inductive coding approach was used for this purpose. Using this approach, research findings emanate from the raw data when the transcripts are interpreted to identify recurrent themes and categories. This activity was carried out in Microsoft Word itself. A preliminary coding was done by repeated examination of the transcripts of the five focus group discussions. First, the main themes discussed in the focus group discussion were identified. Thereafter, the categories of concepts within those themes were identified. The sub-themes identified were fed into the questionnaire for the quantitative survey.

All quantitative analyses emanating from the online survey were conducted using Stata 17. To analyse the determinants of the shift in consumption, we used binary logistic regression.

This was done since the outcome variable ‘shift in consumption’ is a binary variable with shift (i.e. increase/decrease) in consumption over the last five years taking the value 1 and no shift (i.e. consumption levels remaining constant) taking the value 0. The same can be represented as the equation given below.

$${\rm{Y = }}{{\rm{\beta }}_0} + {{\rm{\beta }}_1}{\rm{X + \varepsilon }}$$

Y is the outcome variable of interest which is “Shift in consumption”. X consists of all independent variables as explained in Table 1 of the earlier section. This exercise enables us to arrive at the variables actually influencing the switch to millet consumption.

To strengthen the findings and identify the main channels, we further conducted exploratory factor analysis and confirmatory factor analysis Structural Equation Modelling (SEM). It allows us to account for errors that might have come up in the regression analysis. It also allows us to work with constructs and latent variables thereby providing robust estimates. We conducted exploratory and confirmatory factor analysis to arrive at the constructs and variables loading on them. The subsequent section contains a detailed explanation.

We conducted several tests to determine the fitness of my model. We ascertained absolute fit indices. Root mean square error of approximation (RMSEA) tells us how well the model, with optimally chosen but unknown parameter estimates would fit the population’s covariance matrix [38]. The standardised root mean square residual (SRMR) is the square root of the difference between the residuals of the sample covariance matrix and the hypothesised covariance model with well-fitting models obtaining values less than 0.05 [38]. Values up to 0.08 are deemed acceptable [39]. The Comparative Fit Index (CFI) performs well even with small samples [40]. A value of CFI ≥ 0.95 is indicative of a good fit [39]. These tests helped us decide how robust our findings were.

To analyse the deterrents to trying out millets, we again used binary logistic regression. The outcome variable ‘Willingness to try out millets’ is binary and takes the value of 1 if non consumers are willing to try out millets and 0 if they are not willing to. The same can be represented as the equation given below.

$${{\rm{Y}}^{\rm{W}}} = \,{{\rm{\beta }}^{{{\rm{W}}_0}}} + {{\rm{\beta }}^{{{\rm{W}}_1}}}{{\rm{X}}^{\rm{W}}} + {\rm{\varepsilon }}$$

Y^w^ is the outcome variable of interest which is “Willingness to try out millets”. X^w^ consists of all independent variables as explained in Table 2 of the earlier section. This enabled us to arrive at the variables actually deterring non consumers of millets from trying them out.

## Results

The demographic profile and basic details of consumption habits are provided in Table 3.

**Table 3:** Sample characteristics and profile (n = 875)

Variables	%
**Education level of head of household**	
Graduate	2.8
Post Graduate	27.5
Professional qualification (BE/MBA/MCA/CA etc.	68.9
Doctorate	0.00
**Monthly Income slab of household (Rs.)**	
50,000–75,000	3.3
75,000-100,000	34.9
100,000-150,000	6.8
> 150,00	54.8
**Millet (jowar, bajra, ragi) consumption habits**	
Millet consumers	75.8
Not millet consumers	24.2
Shift in millet consumption in the last 5 years	52.9
**Influencers for millet consumption**	
Been consuming traditionally	19.4
Advised by doctor due to existing health conditions	65.9
Advised by doctor to pre-empt illnesses viz. hypertension, sugar etc.	9.9
Self-initiated measure to pre-empt lifestyle diseases	60.8
Allergic to gluten	72.2
In-store advertisements	2.1
Social media advertising	47.6
Friends and family	51.7
Celebrity endorsements	25.7
To cultivate healthy eating habits in family members	52.6
Concerns over environmental sustainability	55.3
**Deterrents to millet consumption**	
Lack of awareness about health benefits	32.4
Not easily available	62.3
Not very palatable	89.4
Organic variants not readily available	54.2
Branded products not readily available	67.7
Expensive	37.6
Lack of incentives like discounts	29.1
Preference for exotic products like quinoa	23.7
Family members do not prefer eating millet	69.9
Inadequate cooking skills	75.3

Approximately 75% of the respondents are working professionals and nearly 61% have monthly earnings exceeding INR 100,000. Nearly 76% of the respondents consume millets. Interestingly, approximately 53% of the respondents said that they had shifted to millets in the last five years.

### Results of the thematic analysis of focus groups

Content analysis revealed different themes and categories that are explained hereafter. This exercise allowed us to achieve the purpose of conducting this research: (a) understanding the antecedents of the shift to millet consumption and (b) understanding the deterrents that dissuade consumers from making this shift.

### Understanding the antecedents of the shift to millet consumption

Two major themes were identified in the discussion relating to factors facilitating the participants’ shift to millets. One was reasons pertaining to health and the other was the influence of their social networks. Within the first theme, three major categories emerged. The first was a shift on account of their treating doctor’s advice to manage lifestyle diseases such as diabetes and hypertension. One diabetic participant stated “My sugar levels got controlled only after I started eating millet rotis millet rice instead of regular rice. I have also quit maida.” The second was to pre-empt the onset of such diseases in people who were predisposed to them. One respondent stated “My mother and grandfather both have diabetes. As of now, I don’t have it but it I am very likely to get it sooner or later. I have just made some diet and lifestyle related changes in my life to delay its onset. That is how I started eating millets.” The third was a shift in response to a self-initiated health consciousness to stay fit. One participant stated “I like to stay fit. Eating right and exercising are the core principles that I live by.” These are three very different and unrelated factors nested within the broader construct of health and should be treated as separate to obtain a more granular sense of data.

Within the broader theme of health, a couple of other antecedents also came up during discussions. Consumers who were intolerant to gluten recorded better health outcomes owing to their shift to millets. A participant stated “I am allergic to wheat. I eat millet rotis for dinner and rice for lunch.” For a few consumers, inculcating healthy eating among children was a reason for shifting to millets. One participant stated “How will I make by children eat healthy if I myself don’t eat healthy?”

Within the second theme of social networks, two broad categories emerged. One was friends and family and the other one was social media that included social networking sites as well as messaging apps. One participant stated “I see a deluge of millet recipes on social media. I get tempted to try them. They are novel.” A few consumers also talked about getting influenced by celebrity endorsements in favor of millets and hence shifting to millets. One participant stated “I like the millet recipe videos that Shilpa Shetty posts. I often try them. They are simple and tasty, and of course, nutritious.”

We incorporated these categories into our questionnaire to obtain a deeper understanding of the factors driving people’s shift to millets. The table on measures details out the same.

### Understanding the deterrents that dissuade consumers from making this shift

Access, affordability, and perception emerged as the major themes here. Within the theme of access, the following categories emerged: limited availability and lack of trustworthy options such as organic and branded varieties. Within the theme of affordability, the following categories emerged: high price point and lack of extrinsic motivation such as discounts, cashbacks and freebies. On participant stated “Most millets are almost double the price of atta and rice. Plus, there are hardly any offers on buying them.” Within the theme of perception, the following categories emerged: perception of millets as inferior and hence preference for more premium exotic options, perception of millets being unpalatable and perception of millets being difficult to cook. One participant stated “If I have to pay so much, I’d rather buy quinoa.” Another participant stated “I don’t think I cook it so well because no one in my house likes it.” Another participant stated “My cook refuses to make millet rotis because they take longer to prepare and are harder as well.” We incorporated these categories into our questionnaire to obtain a deeper understanding of the factors deterring people from shifting to millets. The table on measures details out the same.

### Results of the quantitative analysis

#### Determinants of shift in millet consumption

Table 4 below provides the results of the regression analysis run for testing this model. We found good correspondence between the dependent variable “shift in consumption towards millets” and independent variables defining “pre-empting health conditions,” “self-initiated health consciousness,” “social media influence” and “social network” at 1% level of significance. This points out to the rise in health consciousness among the urban affluent population. Even among these variables, self-initiated health consciousness and social networks in the form of friends and family seem to have a larger effect on the shift in favor of millets consumption. People with self-initiated health consciousness are 9 times more likely to switch to millets. Respondents influenced more by their social network are 37 times more likely to shift to millets compared to those who are not. This brings forth the fact that health [41] and dietary habits [42] have become an important talking point among people even in their social circles.

**Table 4:** Determinants of shift in consumption towards millets

Shift in consumption	Odds Ratio	Std. Err.	z	P>|z|	[95% Conf. Interval]	
Traditional	1.12	0.32	0.39	0.696	0.633	1.982
Existing health	0.68	0.2	-1.28	0.202	0.385	1.222
Gluten	0.36	0.16	-2.2	0.028	0.152	0.898
Pre-empt health	0.09	0.04	-5.25	0.000**	0.038	0.227
Self-health	9.31	3.48	5.97	0.000**	4.477	19.375
Advertisement	2.43	2.6	0.83	0.408	0.297	19.897
Social media	3.39	1.01	4.1	0.000**	1.892	6.105
Friends family	37.16	11.99	11.2	0.000**	19.745	69.95
Celebrity end	1.52	0.47	1.38	0.168	0.835	2.8
Habit formation	1.4	0.41	1.14	0.253	0.785	2.498
Environmental	1.7	0.5	1.79	0.073	0.951	3.039
_cons	0.07	0.02	-8.91	0	0.044	0.136

Here, the switch does not have a bearing on the quantum of consumption. It simply means that someone who was not eating millets in any form five years back has today included them in their diet. Adding controls for income and education individually and jointly does not alter the results of the analysis. Interactions between health and education and health and income were also not found to be significant either.

**Fig. 1:**
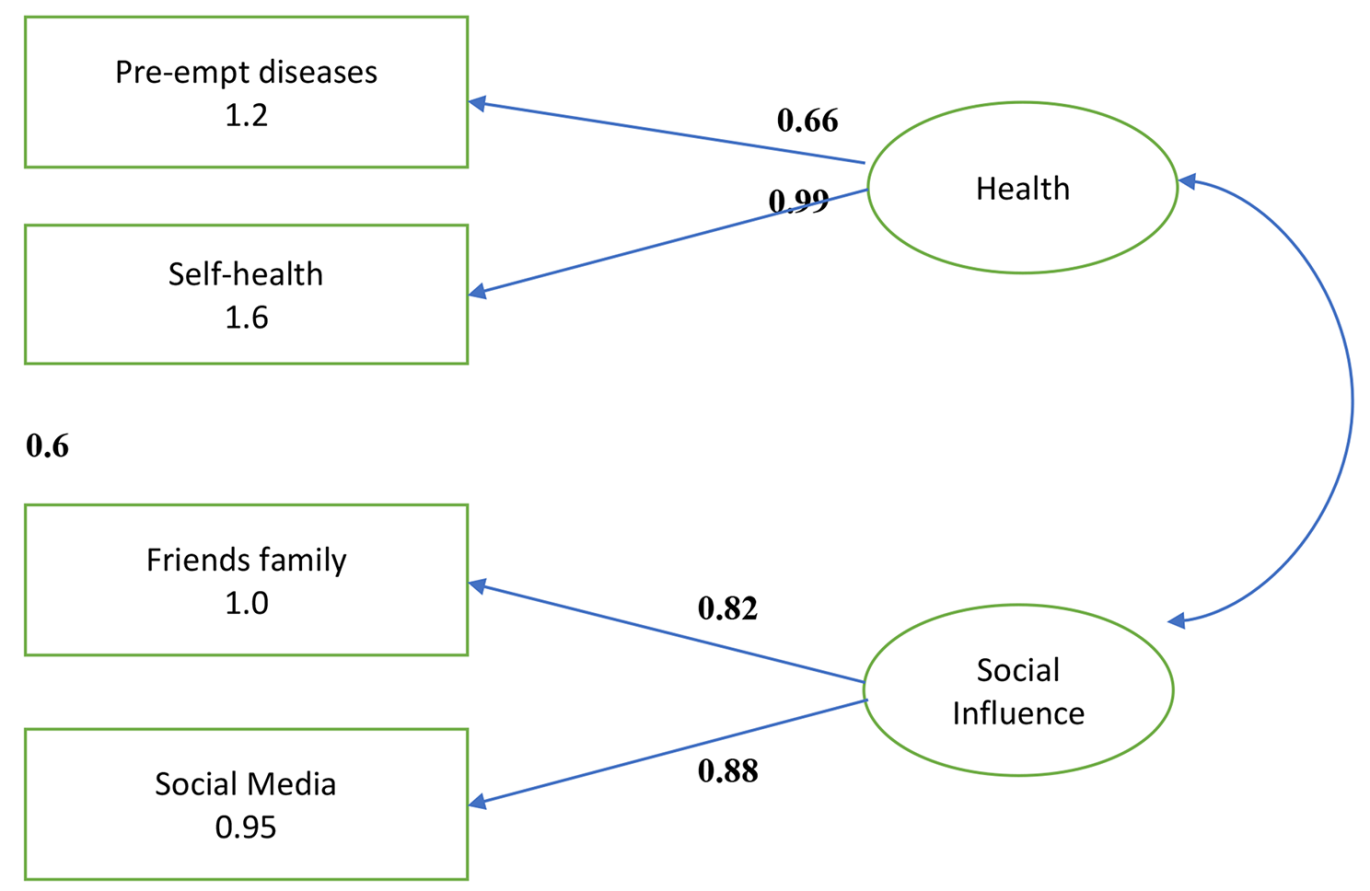
Path diagram for determinants of shift in consumption of millets

We ran Structural Equation Modelling in Stata to further analyse the results obtained through regression (refer to the path diagram in Fig. 1). First, principal component analysis was conducted. Three factors whose Eigenvalues exceeded 1 were retained in the analysis. The three factors or constructs have been explained further in this section. Orthogonal rotation was performed because all 3 factors are independent of each other. Thereafter they were sorted so that factor loadings were obtained for each of the ten variables. These factors or constructs are latent and comprise of measurable variables. The variables “self-health” and “pre-empt diseases” load on Factor 1 with factor loadings of 0.99 and 0.66 respectively. These are health related variables. Hence, we name this construct as Health. As the variables “habit” and “environmentally conscious” also load, albeit weakly, on Factor 1 and do not fit into the health construct, they have been dropped. The variables “friends & family” and “social media” load well on Factor 2, taking values of 0.82 and 0.88 respectively. This factor has been named Social influence and is a behavioural construct of awareness. The variable “advertising” loads on Factor 3 with a factor loading of 0.74. However, it is a single variable construct, and it has been dropped from the study. As is evident from the path diagram, the covariance between the two constructs is less than that between the construct and its variables which means that factor analysis has been done well. The covariance between health and social influence is 0.6. Factor loadings reveal that health and social influence are the most critical constructs leading the shift to millets consumption. This is in line with findings of studies on organic food that show that consumers have shifted to organic food because (i) they perceive them to be healthier and more nutritious [43, 44] and (ii) they are influenced by their social environment and peer influence [45, 46].

The RMSEA statistic figure in our model is 0.073 and is within the acceptable range for a fair model fit [47]. Our model has an SRMR value of 0.01 and is hence a good fit. The CFI statistic figure for the model is 0.997, indicating a good fit.

All the three goodness of fit tests indicate that the model fit is good. The findings from SEM analysis corroborate our findings from Logit regression, thereby lending support to the study findings.

### Deterrents for willingness to try out millets

Table 5 below provides the results of the regression analysis run for testing this model. Lack of awareness, lack of easy availability, high prices, lack of branded products, lack of attractive promotional cashbacks and discounts and family aversion to switching to millets emerge as significant deterrents to trying out millets. Even among these variables, lack of availability of branded millets and the absence of cashbacks or discounts seem to have the strongest effect on deterring consumers from trying out millets as is evident in the table. Adding controls for income and education to the analysis did not alter the results.

**Table 5:** Deterrents for willingness to try out millets

Willingness to try millets	Odds Ratio	Std. Err.	z	P>|z|	[95% Conf. Interval]	
Awareness	0.03	0.02	-4.57	0.000**	0.007	0.145
Availability	0.02	0.01	-4.08	0.000**	0.003	0.134
Palatability	2.03	1.46	0.98	0.325	0.495	8.323
Organic availability	0.71	0.54	-0.45	0.656	0.158	3.186
Branded availability	2534.53	3894.74	5.1	0.000**	124.703	51513.06
Expensive	0	0	-3.82	0.000**	0	0.083
Cashback discount	405.65	550.87	4.42	0.000**	28.327	5808.841
Exotic	65.64	87.16	3.15	0.002	4.864	886.012
Family averse	0	0	-6.4	0.000**	0	0.005
Inadequate cooking skill	0.4	0.29	-1.25	0.212	0.096	1.679
_cons	0.03	0.05	-2.24	0.025	0.001	0.659

## Discussion

The primary aim of this study was to understand the determinants of the shift to millet consumption among urban affluent consumers. As the data reveal, 52.6% of the respondents recorded an increase in millet consumption over the last 5 years. Health consciousness and social networks seem to be the major factors in bringing about this shift. Millets are rapidly catching up as a preferred choice of food grains among urban affluent consumers. This is contrary to the prevalent notion that millets are a rural, low income and low class food. This can be explained by the fact that India is extremely heterogenous in terms of social, economic, and cultural indicators as well as culinary practices and diet preferences [48]. Multiple diets co-exist within the broader milieu. Urban consumers are willing to experiment with these grains and are looking for new product variants. Through this study, we have sought to explain the reasons for this shift in consumption as well as the deterrents to shifting to millets.

With the rising incidence of health problems, lifestyle diseases and rising consciousness about health and fitness, the concept of food healthiness is gaining ground. As increasing scientific evidence is emerging about the healthiness of millets and awareness about them is spreading, urban consumers are making a switch to millets and millet-based products. Their social network and the new age social media are important influencers. People today have more information at their disposal today at the click of a button. The idea of the healthfulness of dietary intake has been gaining ground rapidly, especially among the more affluent and educated households [49]. These factors have led to a favourable response of urban consumers to millets. They also find support in the Health Belief Model as well. People who are predisposed to medical conditions and those who are more health conscious are more likely to switch to millet consumption. Contrary to our expectations, such results were not observed for people who were already diagnosed with lifestyle diseases. This is contrary to logic where one might switch to millets in order to mitigate further health damage. However, one possible explanation is that they are already taking medication for their medical conditions and observing restraint in diet apart from making other lifestyle changes. As a result, they are not taking extreme steps to alter their basic food regime. These insights were obtained during the personal interviews and focused group discussions. Awareness, availability and product promotions are critical to enabling non-consumers to at least try out millets.

Given that health is a critical antecedent for consumers’ shift to millets, care must be taken to retain the health aspect while developing new product variants and recipes. The addition of sugar, salt, saturated fats, refined flour, and preservatives must be avoided or minimised because of the proven adverse health hazards they pose as a consequence of overuse and industrial processing [50, 51]. Such ultra-processed and processed foods that are loaded with excess sugar, salt and oils not just provide empty calories [52] but also displace more nutrient dense foods [53]. This results in overfed but undernourished consumers. Consumers are much more aware and read the product label very carefully while making their purchase [54]. Product development is challenging because the health aspect and palatability both need to be carefully balanced. However, it can be rewarding because there is an enormous market to be tapped. The findings of this study also show that consumers would be willing to try out millets if branded millets can be made available easily and be offered discounts or cashbacks to at least get consumers to try out millets. More awareness about their health benefits needs to be generated. Better labelling of packages to increase awareness and building trust would be a step closer to increasing consumer acceptance of the product [55].

For food companies, customer engagement is crucial. Many customers might be potential first time consumers of the product [56]. The presence of nutrition claims influences consumers’ purchase intentions [57]. Hence, companies need to be mindful of how and what messages they want to convey through their packaging and advertisements. These heuristics serve as critical antecedents for consumer choices [58].

As previously discussed, this study has critical implications for public policy. Messaging around the health benefits or behaviour change mechanisms needs to be tailored around health conscious individuals. Those who are not health conscious but are susceptible to DR-NCDs owing to their predisposition, lifestyle or other factors need to be made aware and conscious. Awareness campaigns similar to egg and milk consumption pioneered by the National Egg Co-ordination Committee (NECC) and AMUL may be brought out to extol the goodness of these grains and encourage people to include them in their regular diet.

As discussed earlier, India, like the rest of the world, is seeing a surge in cases of hypertension, obesity and diabetes. Chronic diseases related to overnutrition are on the way to becoming a public health problem [59]. The prevalence of DR-NCDs is higher in more affluent and developed states of the country [60]. Impaired glucose tolerance, type-II diabetes mellitus and several chronic and non-communicable diseases have become very rampant in the last few years [61]. Furthermore, micronutrient deficiency continues to pose a severe public health challenge with associated economic costs of 0.8 to 2.4% of the total GDP [62]. It is more rampant among women and children [63] and impacts children’s functional, physical and cognitive performance [64]. Obese women are twice as likely to spend more money on their health than normal weight women. Overweight women are twice as likely to spend a higher proportion of their total household expenditure on their health than normal weight women [65]. This begs the question: how do we mitigate this health hazard? Changes in diet in favour of healthy wholesome food items are key to any such solution. To mitigate these health challenges, it is important for policymakers to address issues around the access and price of millets and their derivatives. The less affluent largely consume wheat and rice obtained at a highly subsidised price through the Public Distribution System (PDS) [66]. They may not have enough awareness and/or money to consume millets regularly. For such sections of the society, the issues of awareness, cost and access hold salience. To enable equitable access to them, key stakeholders such as the government and private players need to formulate a favourable supply response.

Since DR-NCDs are a global problem, the implications of this study are also for a global audience. A total of 1.9 billion adults were obese or overweight in 2016 [67] while nearly 3 million deaths occur annually due to obesity or related diseases [68]. The cost of obesity alone is likely to be between 0.7% and 2.8% of a nation’s total health care costs [69]. In countries with a higher prevalence of obesity such as the US, these expenses are likely to double every ten years and will most likely account for 16–18% of total health care expenses by 2030 [70]. These predictions warrant urgent policy action to tackle this ever growing pandemic of DR-NCDs. While the production of millets is largely confined to Africa and Asia, globalisation can ensure their availability across the world. That is where the role of the governments and market comes in. The health and policy implications that hold good for India would most likely hold good for the rest of the world too. Studies performed in Europe and the US on other kinds of foods such as whole grains suggest that their increased consumption mitigates the risk of cancer, diabetes, obesity, and cardiovascular diseases and that awareness and availability of good nutritious options can enhance their consumption [71]. Likewise, studies on the health implications of organic foods [72] performed in the West hold good globally. Learnings from these contexts serve well even in India. Likewise, it can be safely assumed that the findings of this present study hold good for India and for any other part of the world as well. Awareness about health benefits and easy availability hold the key to shifting to the consumption of millets.

This study has a few limitations. As discussed earlier, we conducted purposive sampling for this study. Future research needs to cover more heterogeneous samples to generalise the results quantitatively. It is possible that those who were more likely to participate in the survey were also more likely to be concerned about their health. However, we were unable to control for selection bias due to limited data. Furthermore, we included many products within the larger nomenclature of millets. Further research is needed to deeply examine which product variants consumers are switching to. For millet consumption to bring about a substantial change in consumers’ health parameters, they should be consumed in totality in their raw and purest form which is minimally processed. Eating millet wafers and chips will not make people healthier. Hence, it needs to be understood what forms of millets people consume. Communication of their health benefits also needs to be tailored in a way as to disseminate the right kind of unambiguous message. Seasonality in consumption has not been factored in this study and can be explored in future studies. Future studies can also focus on studying millet wise trends in consumption focusing on traditionally non millet eating belts of the country.

## Conclusion

Millets hold much promise in tackling the dual challenges of malnourishment and environmental sustainability. Several past studies provide evidence to support this claim. This study shows that urban affluent consumers see value in them. They are aware of their health benefits and are therefore transitioning to millets. Marketers can act as catalysts in seizing this market opportunity. At the policy level, promoting the consumption of millets and incentivizing their cultivation and value addition to bring these nutri-cereals to a larger population would be in the larger public interest and would be a step towards easing the burden of rising health costs.

### Ethical disclosure

The surveys were conducted according to established ethical guidelines, and informed consent was obtained from the participants in the online surveys as well as the formative field work. The study complies with all regulations.

### Electronic supplementary material

Below is the link to the electronic supplementary material.

Supplementary Material 1

## Data Availability

The datasets used and/or analysed during the current study are available from the corresponding author upon reasonable request.

## Footnotes

1 India is the largest millet producer in the world. Her Pearl Millet production accounted for 40.51% followed by Sorghum 8.09% of the global production of Millets in 2020. Six Indian states namely, Rajasthan, Karnataka, Maharashtra, Uttar Pradesh, Haryana and Gujarat contribute to more than 83% share in total millet production of the country. Bajra, jowar and ragi hold the largest share in India’s total millet production (APEDA, 2023).

2 Social desirability bias arises when respondents give answers they feel are aligned with their social norms. This bias is assumed to be more pronounced in face-to-face personal interviews than in online surveys because of the presence of an interviewer in the former case (75). Hypothetical bias refers to discrepancy between what respondents say in a hypothetical survey and how they actually behave in a real life purchase situation. As the responses were completely anonymised, the chances of hypothetical bias reduce drastically.

## Abbreviations

DR-NCDs: Diet related non communicable diseases
SDGs: Sustainable Development Goals
USDA: United States Department of Agriculture
BMI: Basal Metabolic Index
NT: Nutrition transition
SEM: Structural Equation Modelling
RMSEA: Root mean square error of approximation
SRMR: Standardised root mean square residual
CFI: Comparative Fit Index
NECC: National Egg Co-ordination Committee
GDP: Gross Domestic Product
PDS: Public Distribution scheme
WHO: World Health Organisation
