# Supplementary material for: Transitioning diets: a mixed methods study on factors affecting inclusion of millets in the urban population
Source: BMC Public Health. 2023 Oct 13;23:2003. doi: 10.1186/s12889-023-16872-5 (PMC10576316; doi:10.1186/s12889-023-16872-5)
Supplement: Supplementary file 1 — Supplementary Material 1 [file 12889_2023_16872_MOESM1_ESM.docx]

**Appendix 1: Discussion guide for Focus group**

- 1. Brief personal details about participants including their personal and professional background
  2. Which regions of India the participants belonged to?
  3. Whether they knew about millets?
  4. Whether millets had been a part of their diet while they were growing up? If so, which ones, and if no, then which cereals were staple for them?
  5. Their current food basket composition
  6. The status of their/family members’ health
  7. For non-native consumers, what was the source(s) of information/knowledge about millets?
  8. Why they were consuming/not consuming millets? If consuming, then what products? Some details about them
  9. What was their outlook for the future with regards to their consumption of millets

**Appendix 2: Outline of the online questionnaire**

1. Do you consume millets (jowar/bajra/ragi)?
2. If no, would you be willing to try them out?
3. On a scale of 1-5, how would you rate your preference for millets?

Not at all preferred – Very much preferred

1. Your reasons for consuming millets:
   - 1. Been traditionally eating them
     2. Advised by doctor due to existing health conditions (viz. hypertension, diabetes, obesity)
     3. Allergic to gluten
     4. Advised by doctor to pre-empt health conditions viz BP, sugar etc
     5. Self-initiated measure to pre-empt lifestyle diseases
     6. In-store advertisements and inducements
     7. Social media advertising
     8. Friends/relatives act as influencers
     9. Celebrity endorsements
     10. To cultivate healthy eating habits in family, esp. in children
     11. Environmentally sustainable
2. How would you rate your current consumption of millets when compared to five years ago?
3. Increased
4. Decreased
5. Remained constant
6. Frequency of consumption
7. twice or more every week
8. at least 4 times a month
9. occasionally
10. never
11. Your reasons for not consuming millets:
12. Not much awareness about their health benefits
13. Not easily available
14. Not very palatable
15. Organic products not easily available
16. Branded products not easily available
17. Expensive
18. Lack of promotional offers such as cashbacks/discounts/free gifts
19. Higher preference to exotic healthier options, such as quinoa
20. Reluctance of one or more family members to consume millets
21. Inadequate cooking skills
22. Highest education of any family member?
23. Graduate
24. Post-graduate
25. Professional graduate, e.g. Engineer, Doctor, CA, MBA etc.
26. Monthly income range of household (in INR)
27. 50,000 – 75,000
28. 75,000 – 100,000
29. 100,000 – 150,000
30. >150,000
